# Renormalization group theory of molecular dynamics

**DOI:** 10.1038/s41598-021-85286-3

**Published:** 2021-03-16

**Authors:** Daiji Ichishima, Yuya Matsumura

**Affiliations:** Technology Research Center, Sumitomo Heavy Industries, Ltd., 19 Natsushima-cho, Yokosuka-shi, Kanagawa 237-8555 Japan

**Keywords:** Applied physics, Statistical physics, thermodynamics and nonlinear dynamics, Mathematics and computing

## Abstract

Large scale computation by molecular dynamics (MD) method is often challenging or even impractical due to its computational cost, in spite of its wide applications in a variety of fields. Although the recent advancement in parallel computing and introduction of coarse-graining methods have enabled large scale calculations, macroscopic analyses are still not realizable. Here, we present renormalized molecular dynamics (RMD), a renormalization group of MD in thermal equilibrium derived by using the Migdal–Kadanoff approximation. The RMD method improves the computational efficiency drastically while retaining the advantage of MD. The computational efficiency is improved by a factor of $2^{n(D+1)}$ over conventional MD where *D* is the spatial dimension and *n* is the number of applied renormalization transforms. We verify RMD by conducting two simulations; melting of an aluminum slab and collision of aluminum spheres. Both problems show that the expectation values of physical quantities are in good agreement after the renormalization, whereas the consumption time is reduced as expected. To observe behavior of RMD near the critical point, the critical exponent of the Lennard-Jones potential is extracted by calculating specific heat on the mesoscale. The critical exponent is obtained as $\nu =0.63\pm 0.01$. In addition, the renormalization group of dissipative particle dynamics (DPD) is derived. Renormalized DPD is equivalent to RMD in isothermal systems under the condition such that Deborah number $De\ll 1$.

## Introduction

There are a vast number of modern applications where phase transition or interface plays an important role such as laser annealing and 3D printing. It has long been desired to perform computational experiments of such macroscopic, multiphysics problems, which are often challenging for continuum approaches. There has also been a strong interest in a method capable of seamlessly simulating from microscale, mesoscale to even a continuum regime. The only method that satisfies these demands in principle is molecular dynamics (MD)^1,2^, and it has been adopted in a wide variety of fields such as nanostructures^3^ and biochemistry^4,5^.

A major shortcoming of MD is that it requires a vast amount of computational resources especially for solving large systems. Recently, advancement in hardware, parallel computing methods as well as graphical processing units (GP-GPUs) have drastically increased the simulation scale achievable^6–9^, but a majority of MD studies are still limited to the orders of micrometers and nanoseconds, and macroscopic analyses are often even impractical. Several solutions have been developed to overcome this limitation. In the fields of biochemistry and biophysics, common techniques are coarse-graining^10–12^ and enhanced sampling methods^13–15^. Another approach, often adopted for nanofluids and nanostructures, is hybrid methods of atomic-continuum domains^16,17^.

Here we propose a new approach to expand the applicability of conventional MD. P. W. Anderson suggested that calculations in arbitrary scale are possible by applying the renormalization group (RNG) theory to transport phenomenon^18^ [pp. 212]. If the Hamiltonian describing MD is at a fixed point, following the RNG transformation, a calculation in arbitrary scale can be conducted with the same number of particles. This idea coarse-grains the entire domain uniformly so that a drastic improvement on the computational efficiency may be achieved in mesoscale or even macroscopic systems, while retaining the aforementioned advantages of MD. The RNG of elastic bodies has been constructed for coarse-grained MD^19–21^ [Sect. 13.5]. Faccioli et. al adopted the idea of RNG to decouple the short-time fluctuations and the long-term dynamics of the molecular system described by the Langevin equation^22,23^ (i.e. dissipative dynamics), but the same idea cannot be applied to MD since energy of a particle is not conserved. To the best of our knowledge, the RNG for MD has not yet been constructed.

In the current work, we derive the RNG by using the Migdal–Kadanoff approximation^21,24^ [Sect. 16.6.4]. The obtained RNG for MD, or renormalized molecular dynamics (RMD), is verified by two test problems; melting of an aluminum slab and collision of aluminum spheres. We then discuss the computational efficiency of RMD over conventional MD and the similarity of physical phenomena after the renormalization. Furthermore, to observe behavior of RMD near the critical point, we calculate specific heat at constant volume of the Lennard-Jones fluid and investigate the finite size scaling. In addition, the RNG of dissipative particle dynamics (DPD) is derived and the relationship between RMD and DPD is discussed.

### Renormalization of the Hamiltonian

The total Hamiltonian *H* with *N* particles is given as:

1 $$\begin{aligned} H = \sum _{j=1}^{N} \left\{ \frac{{p}_j^2}{2m}+ \frac{1}{2}\sum _{k\ne j}^N\phi ({\varvec{r}}_j-{\varvec{r}}_k)\right\} \end{aligned}$$

where ${\varvec{p}}$ is momentum of a particle, ${\varvec{r}}$ is position, *m* is mass and $\phi$ is interatomic interaction. We consider interaction of the following form:

2 $$\begin{aligned} \phi (r)=\epsilon f\Big (\frac{r-r_o}{\sigma }\Big ) \end{aligned}$$

where $\epsilon$ is potential depth, $\sigma$ is particle diameter and $r_o$ is position of the minimum of the potential. Examples of such potentials are the well-known Morse and Lennard-Jones (LJ) potential. The renormalized Hamiltonian is obtained by integrating a part of the distribution function and rescaling the phase space. If the number of particles is reduced whilst keeping the distribution function invariant, the observables corresponding to the ensemble average in equilibrium do not change.

### Coarse-graining of an atomic chain

We start coarse-graining from the Hamiltonian of an atomic chain of length *L*. A distribution function of the Canonical ensemble with *N* particles is given as:

3 $$\begin{aligned} \Xi (\beta ) = \frac{1}{N!h^{3N}} \int \prod _{i=1}^{N} d {\varvec{p}}_i d {\varvec{r}}_i e^{-\beta H} \end{aligned}$$

where *h* is the Planck constant, $\beta ={1/(k_B T})$, *T* is temperature and $k_B$ is the Boltzmann constant. The coarse-graining of the atomic chain is obtained by removing a degree of freedom of a particle *j* locating at midpoint of particles *i* and *k*. The potential on the particle *j* formed by the nearest neighbors can be written as:

$$\begin{aligned} {\phi }({\varvec{r}}_i-{\varvec{r}}_j)+{\phi }({\varvec{r}}_j-{\varvec{r}}_k) ={\phi }\Big (\frac{{\varvec{r}}_i-{\varvec{r}}_k}{2}+\frac{{\varvec{r}}_i+{\varvec{r}}_k-2{\varvec{r}}_j}{2}\Big )+ {\phi }\Big (\frac{{\varvec{r}}_i-{\varvec{r}}_k}{2}-\frac{{\varvec{r}}_i+{\varvec{r}}_k-2{\varvec{r}}_j}{2}\Big ) \end{aligned}$$

Taylor series expansion on ${{\varvec{r}}_i-{\varvec{r}}_k\over 2}$ gives:

4 $$\begin{aligned} {\phi }({\varvec{r}}_i-{\varvec{r}}_j)+{\phi }({\varvec{r}}_j-{\varvec{r}}_k) = 2{\phi }\Big (\frac{{\varvec{r}}_i-{\varvec{r}}_k}{2}\Big )+2\sum _{l=1}^\infty \frac{\sigma ^{2l}}{ (2l)!}{\phi }^{(2l)}\Big (\frac{{\varvec{r}}_i-{\varvec{r}}_k}{2}\Big )x_j^{2l} \end{aligned}$$

Here, $x_j$ and $\phi ^{(2l)}$ are defined as:

5 $$\begin{aligned}&x_j =\frac{1}{2\sigma r_{i,k}}({\varvec{r}}_i-{\varvec{r}}_k)\cdot ({\varvec{r}}_i+{\varvec{r}}_k-2{\varvec{r}}_j) \end{aligned}$$

6 $$\begin{aligned}&\phi ^{(2l)}\Big ({{\varvec{r}}_i-{\varvec{r}}_k\over 2}\Big )={\partial ^{2l}\phi (r)\over \partial r^{2l}}\Biggl |_{ r={| {\varvec{r}}_i-{\varvec{r}}_k |\over 2}} \end{aligned}$$

We define $z_{2l}$ as a root of $\phi ^{(2l)}(r)$. For the Morse and LJ potentials, $\phi ^{(2l)}(r=z_{2l})$ are given as:

7 $$\begin{aligned} \phi ^{(2l)}(z_{2l}) = \Biggl \{\begin{array}{ll} {\epsilon \over \sigma ^{2l}}\bigl [2^{2l}e^{-(z_{2l}-r_o)/\sigma }-2\bigr ]e^{-(z_{2l}-r_o)/\sigma } &{} \quad \text {for Morse potential}\\ {4\epsilon \over \sigma ^{2l}}\bigl [ {(11+2l)!\over 11!}\bigl ({\sigma \over z_{2l}}\Bigr )^{6} -{(5+2l)!\over 5!} \bigr ]\bigl ({\sigma \over z_{2l}}\bigr )^{(6+2l)} &{}\quad \text {for LJ potential} \end{array} \end{aligned}$$

Therefore, having $\phi ^{(2l)}(z_{2l})=0$ yields:

8 $$\begin{aligned} z_{2l}= {}&\Biggl \{ \begin{array}{ll} \sigma (2l-1) \log _e2+r_o &{} \quad \text {for Morse potential}\\ \sigma \bigl [ \frac{(11+2l)!5!}{(5+2l)!11!} \bigr ]^{1\over 6} &{}\quad \text {for LJ potential} \end{array} \end{aligned}$$

Next step is the integration for variables ${\varvec{p}}_j$ and ${\varvec{r}}_j$. Using Eq. (4) gives:

9 $$\begin{aligned}&\frac{1}{h^3}\int _{-\infty }^{\infty }d^3p_j \int ^{{\Delta L\over 2}}_{-{\Delta L\over 2}}d^3r_j e^{-\beta \Big \{\frac{{p}^2_i}{2m}+\frac{{p}^2_j}{2m}+\frac{{p}^2_k}{2m}+{\phi }({\varvec{r}}_i-{\varvec{r}}_j)+{\phi }({\varvec{r}}_j-{\varvec{r}}_k)\Big \}} \nonumber \\&\quad =v_f({\varvec{r}}_{i,k}) e^{-\beta \Big \{\frac{p^2_i}{2m}+\frac{p^2_k}{2m}+2{\phi }\left( \frac{{\varvec{r}}_{i,k}}{2}\right) -k_BT\log {\frac{(2\pi mk_BT)^\frac{3}{2}}{h^3}} \Big \} } \end{aligned}$$

10 $$\begin{aligned}&v_f({\varvec{r}}_{i,k})= \sigma \Delta L^2 \int ^{\frac{\Delta L}{2\sigma }}_{-\frac{\Delta L}{2\sigma }} dx_j e^{-2\beta \sum _{l=1}^\infty \frac{\sigma ^{2l}}{2l!}{\phi }^{(2l)}\left( \frac{{\varvec{r}}_{i,k}}{2}\right) x_j^{2l}} \end{aligned}$$

where $\Delta L=L/N$ and $v_f$ is the free volume^25^ [p.62,Sect. 4.2]. The most challenging part of this derivation would be the integration of the free volume. As $\frac{r_{i,k}}{2}$ increases, a sign of $\phi ^{(2l)}(r_{i,k}/2)$ changes from positive to negative at $z_{2l}$, then $\phi ^{(2l)}(r_{i,k}/2)$, after taking minimum values, asymptotically approaches to zero. In the range of $\phi ^{(2l)}(r_{i,k}/2)\le 0$, the integral Eq. (10) diverges at a limit of $\Delta L/(2\sigma )\rightarrow \infty$. It is required to have $v_f\le \Delta L^3$ (i.e. the collective entropy), so $\phi ^{(2l)}(r_{i,k}/2)$ should be adjusted to zero if $\phi ^{(2l)}(r_{i,k}/2)\le 0$. By this manipulation, a leading term in the range of $\phi ^{(2l)}\le 0$ would become a term of $\sim \phi ^{(2(l+1))}x^{2(l+1)}_j$.

As an example, we discuss an approximation when the summation is cut off at $l=2$. Eq. (10) can be written as:

$$\begin{aligned} v_f({\varvec{r}}_{i,k})\approx \sigma \Delta L^2 \int dx_j e^{-2\beta \{\frac{\sigma ^{2}}{2!}{\phi }^{(2)}\left( \frac{{\varvec{r}}_{i,k}}{2}\right) x_j^{2} + \frac{\sigma ^{4}}{4!}{\phi }^{(4)}\left( \frac{{\varvec{r}}_{i,k}}{2}\right) x_j^{4}\}} \end{aligned}$$

We have $\phi ^{(2)}>0$ in the range of $0<{|{\varvec{r}}_{ij}|\over 2}<z_2$. Ignoring a term of $x^4_j$, we obtain:

$$\begin{aligned} v_f({\varvec{r}}_{i,k})\approx \sigma \Delta L^2 \int dx_j e^{-2\beta \frac{\sigma ^{2}}{2!}{\phi }^{(2)}\left( \frac{{\varvec{r}}_{i,k}}{2}\right) x_j^{2}} \end{aligned}$$

Similarly, we have $\phi ^{(2)}<0$ and $\phi ^{(4)}>0$ in the range of $z_2<{|{\varvec{r}}_{ij}|\over 2}<z_4$. Thus, $\phi ^{(2)}$ is adjusted to zero and we have:

$$\begin{aligned} v_f({\varvec{r}}_{i,k})\approx \sigma \Delta L^2 \int dx_j e^{-2\beta \frac{\sigma ^{4}}{4!}{\phi }^{(4)}\left( \frac{{\varvec{r}}_{i,k}}{2}\right) x_j^{4}} \end{aligned}$$

By the approximation described above, at an arbitrary ${\varvec{r}}_{i,j}$, the integral can be rewritten as:

11 $$\begin{aligned}&v_f({\varvec{r}}_{i,k}) \approx \sigma \Delta L^2 \int ^{\frac{\Delta L}{2\sigma }}_{-\frac{\Delta L}{2\sigma }} dx_j e^{-2\beta \frac{\sigma ^{2l}}{(2l)!}{\phi }^{(2l)}\left( \frac{{\varvec{r}}_{i,k}}{2}\right) x_j^{2l}}~ \end{aligned}$$

12 $$\begin{aligned}&z_{2(l-1)}\le {|{\varvec{r}}_{i,k}|\over 2} <z_{2l} , \quad l=1,2,3,\ldots \end{aligned}$$

Here, $z_0=0$ and always $z_{2(l-1)}<z_{2l}$. Large $x_j$ does not contribute to the integration because $\phi ^{(2l)}\le 0$ does not exist. Therefore, by extending the integration range as $\Delta L/(2\sigma )\rightarrow \infty$, Eq. (11) is reduced to the gamma function. From definition of the gamma function, $\Gamma (l^{-1})=l\int ^\infty _0e^{-x^l}dx$, the integration becomes:

13 $$\begin{aligned} v_f({\varvec{r}}_{i,k}) \approx {}&\Delta L^2 l^{-1}\varGamma \left( \frac{1}{2l}\right) \bigg \{\frac{(2l)!}{2\beta \phi ^{(2l)}(\frac{{\varvec{r}}_{i,k}}{2})}\bigg \}^{\frac{1}{2l}} \end{aligned}$$

14 $$\begin{aligned} z_{2(l-1)}\le&\frac{|{\varvec{r}}_{i,k}|}{2}<z_{2l} \end{aligned}$$

By substituting Eqs. (9) and (13) into Eq. (3), the distribution function of the atomic chain after the coarse-graining can be obtained as:

15 $$\begin{aligned} \Xi (\beta ) = {}&\frac{1}{(N/2)!h^{N/2}} \int \prod _{i=1}^{N/2} d{\varvec{p}}_i d{\varvec{r}}_i e^{-\beta F'} \end{aligned}$$

16 $$\begin{aligned} F'={}&\sum _{i}^{N/2} \bigg \{ \frac{p^2_i}{2m} - {k_BT}\log {\frac{e\{2\pi m k_BT\}^{\frac{3}{2}}}{2Nh^3}} + \frac{1}{2}\sum ^{N/2}_{k\ne i} 2{\phi }\Big (\frac{{\varvec{r}}_i-{\varvec{r}}_k}{2}\Big ) - \frac{k_BT}{2}\sum ^{nn}_{<k,i>}\log {v_f({\varvec{r}}_{i,k})} \bigg \} \end{aligned}$$

where $F'$ is the Helmholtz free energy after the coarse-graining. Hereafter, an apostrophe indicates the coarse-grained property. The second term of the right hand side of Eq. (16) is given by the Stirring approximation: $\log {{1\over N!}\left( {N\over 2}\right) !}\approx -\frac{N}{2}\log \left( \frac{2N}{e}\right)$. The entropy $S'$ and the internal energy $E'$ of the atomic chain after the coarse-graining are:

17 $$\begin{aligned} S'= {}&-\left( \frac{\partial F'}{\partial T}\right) _L \nonumber \\ ={}&k_B\sum _i^{N/2}\bigg \{ {3\over 2} +\log {\frac{e\big (2\pi m k_BT\big )^{3\over 2}}{2Nh^3}} \bigg \} + {k_B\over 2}\sum _i^{N/2}\sum ^{nn}_{<k,i>}\bigg \{s\Big ({{\varvec{r}}_{i,k}\over 2}\Big )+\log {v_f({\varvec{r}}_{i,k})} \bigg \} \end{aligned}$$

18 $$\begin{aligned} E'={}&F'+TS' \nonumber \\ ={}&\sum _i^{N/2}\bigg \{ \frac{p^2_i}{2m} + {1\over 2}\sum ^{N/2}_{k\ne i}2{\phi }\Big ({{\varvec{r}}_{i,k}\over 2}\Big )+{k_BT\over 2}\sum ^{nn}_{<k,i>}s\Big ({{\varvec{r}}_{i,k}\over 2}\Big )+{3k_BT\over 2} \bigg \} \end{aligned}$$

where $s({\varvec{r}})$ is a step function derived from $s({\varvec{r}})=T{\partial \over \partial T}\log v_f$ such that:

19 $$\begin{aligned} s({\varvec{r}})={1\over 2l},~~ z_{2(l-1)}\le |{\varvec{r}}|<z_{2l}. \end{aligned}$$

By the decimation $N\rightarrow N/2$, the coarse-grained Hamiltonian of the atomic chain is represented as:

20 $$\begin{aligned} H'=\sum _i^{N/2}\Big \{\frac{{p}^2_i}{2m} +{1\over 2}\sum ^{N/2}_{k\ne i}{2\phi }\Big ({{\varvec{r}}_{i,k}\over 2}\Big ) +{k_BT\over 2}\sum ^{nn}_{<k,i>}s\Big ({{\varvec{r}}_{i,k}\over 2}\Big ) \bigg \}. \end{aligned}$$

The last term of the right hand side of Eq. (18), ${3\over 2}k_BT$, is removed from Eq. (20) since it disappears by the normalization.

### Coarse-graining of the *D*-dimensional Hamiltonian

In the previous section, we derived the one-dimensional coarse-graining of the atomic chain. Next, the result is extended to the *D*-dimensional Hamiltonian by the Migdal–Kadanoff approximation^21,24^ [Sect. 16.6.4].

First, we discuss the coarse-graining of a two-dimensional Hamiltonian. We assume that the time average of the atomic positions forms a simple (i.e. square, oblique, rectangular and hexagonal) lattice. The lattice with the time averaged positions of atoms maintains the periodicity of a simple lattice, so that the Migdal–Kadanoff approximation can be applied to atomic systems. For convenience, we adopted the square lattice, which is one of the simple lattice, in the following discussion including figures. Nevertheless, the coarse-graining procedure on this section does not lose its generality.

**Figure 1:**
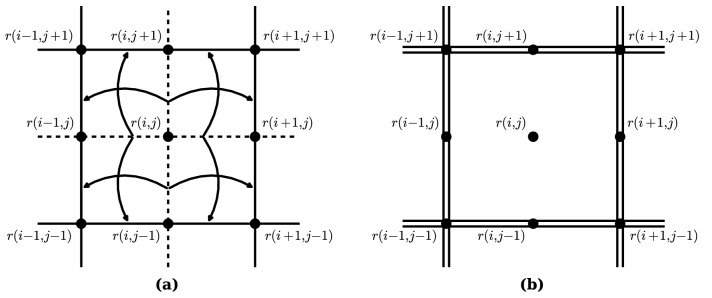
Schematics of the reconstructed interactions of a two-dimensional square lattice by the Migdal–Kadanoff approximation and appearance of atomic chains. Black dot: particles. Solid line: nearest neighbor interactions. Dashed line: interactions to be moved to adjacent interactions. Double line: interactions reconstructed with strength of $2 \epsilon$. (**a**) A two-dimensional square lattice before the Migdal–Kadanoff approximation. (**b**) The square lattice after the Migdal–Kadanoff approximation. Atomic chains appear in the new square lattice.

In the square lattice configuration, a particle at point ${\varvec{r}}_{i,j}$ has four nearest neighbors at ${\varvec{r}}_{i+1,j}$, ${\varvec{r}}_{i-1,j}$, ${\varvec{r}}_{i,j+1}$, ${\varvec{r}}_{i,j-1}$ and four next nearest neighbors at ${\varvec{r}}_{i+1,j-1}$, ${\varvec{r}}_{i-1,j-1}$, ${\varvec{r}}_{i-1,j+1}$, ${\varvec{r}}_{i+1,j+1}$. The particle at ${\varvec{r}}_{i,j}$ can move inside an area of $\Delta L^2$. The atomic arrangement is shown on Fig. 1a. A basic idea of using the Migdal–Kadanoff approximation is to remove an interaction and distribute it into adjacent interactions, so that atomic chains appear and one-dimensional coarse-graining is applicable. For example, let us focus on $\phi ({\varvec{r}}_{i+1,j}-{\varvec{r}}_{i,j})$, one of the nearest neighbor interactions on the particle at ${\varvec{r}}_{i,j}$. By the Migdal–Kadanoff approximation, the interaction is split into half (each has strength of $1/2 \epsilon$) and distributed to adjacent interactions $\phi ({\varvec{r}}_{i+1,j+1}-{\varvec{r}}_{i,j+1})$ and $\phi ({\varvec{r}}_{i+1,j-1}-{\varvec{r}}_{i,j-1})$. This process is applied to all four nearest neighbor interactions on the particle at ${\varvec{r}}_{i,j}$. The same approximation is repeated to adjacent blocks, which contain particles at ${\varvec{r}}_{i+2,j}$, ${\varvec{r}}_{i-2,j}$, ${\varvec{r}}_{i,j+2}$ and ${\varvec{r}}_{i,j-2}$. Therefore, the reconstructed interactions, $\phi ({\varvec{r}}_{i-1,j-1}-{\varvec{r}}_{i,j-1})$, $\phi ({\varvec{r}}_{i,j-1}-{\varvec{r}}_{i+1,j-1})$, $\phi ({\varvec{r}}_{i-1,j-1}-{\varvec{r}}_{i-1,j})$, $\phi ({\varvec{r}}_{i-1,j}-{\varvec{r}}_{i-1,j+1})$, $\phi ({\varvec{r}}_{i-1,j+1}-{\varvec{r}}_{i,j+1})$, $\phi ({\varvec{r}}_{i,j+1}-{\varvec{r}}_{i+1,j+1})$, $\phi ({\varvec{r}}_{i+1,j-1}-{\varvec{r}}_{i+1,j})$ and $\phi ({\varvec{r}}_{i+1,j}-{\varvec{r}}_{i+1,j+1})$, now have a strength of $2\epsilon$. By these manipulations, the Hamiltonian can be rewritten as:

21 $$\begin{aligned} H {}&\approxeq \sum _{i,j}\Big \{ \frac{{p}^2_{i,j}}{2m} +\frac{{p}^2_{i-1,j-1}}{2m} +\frac{{p}^2_{i-1,j+1}}{2m} + \frac{{p^2}_{i+1,j-1}}{2m} + \frac{{p^2}_{i+1,j+1}}{2m} + \frac{{p}^2_{i,j-1}}{2m} + \frac{{p}^2_{i-1,j}}{2m} +\frac{{p}^2_{i,j+1}}{2m} +\frac{{p}^2_{i+1,j}}{2m} \nonumber \\ {}&\quad + 2\phi \Big ({{\varvec{r}}_{i-1,j-1}-{\varvec{r}}_{i,j-1}}\Big ) + 2\phi \Big ({{\varvec{r}}_{i,j-1}-{\varvec{r}}_{i+1,j-1}}\Big ) + 2\phi \Big ({{\varvec{r}}_{i-1,j-1}-{\varvec{r}}_{i-1,j}}\Big ) + 2\phi \Big ({{\varvec{r}}_{i-1,j}-{\varvec{r}}_{i-1,j+1}}\Big ) \nonumber \\ {}&\quad + 2\phi \Big ({{\varvec{r}}_{i-1,j+1}-{\varvec{r}}_{i,j+1}}\Big ) + 2\phi \Big ({{\varvec{r}}_{i,j+1}-{\varvec{r}}_{i+1,j+1}}\Big ) + 2\phi \Big ({{\varvec{r}}_{i+1,j-1}-{\varvec{r}}_{i+1,j}}\Big ) + 2\phi \Big ({{\varvec{r}}_{i+1,j}-{\varvec{r}}_{i+1,j+1}}\Big ) \Big \} \end{aligned}$$

Schematics of the square lattice and the Migdal–Kadanoff approximation is shown on Fig. 1a, b. An integration about ${\varvec{p}}_{i,j}$ is trivial, and the particles at ${\varvec{r}}_{i,j-1},{\varvec{r}}_{i-1,j},{\varvec{r}}_{i,j+1}, {\varvec{r}}_{i+1,j}$ can be considered as particles on the atomic chain. Applying the result of the coarse-graining of the atomic chain Eq. (20) gives:

22 $$\begin{aligned} H \rightarrow H'&= \sum _{i,j}\Big \{ \frac{{p}^2_{i-1,j-1}}{2m} +\frac{{p}^2_{i-1,j+1}}{2m} + \frac{{p^2}_{i+1,j-1}}{2m}+\frac{{p^2}_{i+1,j+1}}{2m}\nonumber \\ {}&\quad +2^2\phi \Big (\frac{{\varvec{r}}_{i-1,j-1}-{\varvec{r}}_{i+1,j-1}}{2}\Big ) +s\Big (\frac{{\varvec{r}}_{i-1,j-1}-{\varvec{r}}_{i+1,j-1}}{2}\Big )k_BT \nonumber \\&\quad +2^2\phi \Big (\frac{{\varvec{r}}_{i-1,j-1}-{\varvec{r}}_{i-1,j+1}}{2}\Big ) +s\Big (\frac{{\varvec{r}}_{i-1,j-1}-{\varvec{r}}_{i-1,j+1}}{2}\Big )k_BT\nonumber \\ {}&\quad + 2^2\phi \Big (\frac{{\varvec{r}}_{i-1,j+1}-{\varvec{r}}_{i+1,j+1}}{2}\Big ) +s\Big (\frac{{\varvec{r}}_{i-1,j+1}-{\varvec{r}}_{i+1,j+1}}{2}\Big )k_BT \nonumber \\&\quad +2^2 \phi \Big (\frac{{\varvec{r}}_{i+1,j-1}-{\varvec{r}}_{i+1,j+1}}{2}\Big ) +s\Big (\frac{{\varvec{r}}_{i+1,j-1}-{\varvec{r}}_{i+1,j+1}}{2}\Big )k_BT\Big \} \nonumber \\ {}&= \sum ^{N/2^2}_{i}\Big \{\frac{{p}^2_{i}}{2m} + {1\over 2}\sum ^{N/2^2}_{k\ne i}2^2\phi \Big (\frac{{\varvec{r}}_{i,k}}{2}\Big ) +{k_BT\over 2}\sum ^{nn}_{<k,i>}s\Big (\frac{{\varvec{r}}_{i,k}}{2}\Big )\Big \} \end{aligned}$$

Finally, by extending the same procedure to the *D*-dimension, the coarse-graining of the *D*-dimensional Hamiltonian is obtained as:

23 $$\begin{aligned} H' ={}&\sum _{i=1}^{N/2^D}\Big \{ \frac{{p}^2_i}{2m} +{1\over 2}\sum ^{N/2^D}_{k\ne i} 2^D\phi \Big (\frac{{\varvec{r}}_{i,k}}{2}\Big )+{k_BT\over 2}\sum _{<k,i>}^{nn}s\Big (\frac{{\varvec{r}}_{i,k}}{2}\Big ) \Big \} \end{aligned}$$

### Derivation of the renormalization transforms

A list of coupling constants, denoted as ${\varvec{K}}$, is given as:

24 $$\begin{aligned} {\varvec{K}} = {}&(\beta ,\epsilon ,r_o,\sigma ,m) \end{aligned}$$

There exist two possible renormalization transforms such that an action $-\beta H$ is at a fixed point. One of such transforms introduces scaling of the space, $L \rightarrow L' =2^{-1}L$. By this transform, the coupling constants are scaled as:

25 $$\begin{aligned} {\varvec{R}}_a({\varvec{K}}) = (2^{-D}\beta ,2^D\epsilon ,r_o,\sigma ,m) \end{aligned}$$

Another transform retains the spatial size *L* invariant, so ${\varvec{K}}$ is scaled as:

26 $$\begin{aligned} {\varvec{R}}_b({\varvec{K}}) = (2^{-D}\beta ,2^D\epsilon ,2r_o,2\sigma ,2^Dm) \end{aligned}$$

Mass density $\rho =N m/L^D$ is at a fixed point for both transforms. However, ${\varvec{R}}_a$ results in the Young modulus *Y* and the speed of sound $c_s$ relevant, since $Y\sim {\epsilon /(\sigma ^2r_o)}$ and $c_s\sim \sqrt{\epsilon r_o^2/(m\sigma ^2)}$ ($\sim \sqrt{k_B T/m}$ for gas)^25^ [Sect. 3.2], whereas ${\varvec{R}}_b$ yields both *Y* and $c_s$ invariant (i.e. at a fixed point). Therefore, we adopted ${\varvec{R}}_b$ for the current work. After the *n* renormalization transforms, the coupling constants ${\varvec{K}}_n$ and the total potential $\Phi _n$ can be written as:

27 $$\begin{aligned} {\varvec{K}}_n ={}&(2^{-nD}\beta ,2^{nD}\epsilon ,2^n r_o,2^n\sigma , 2^{nD} m), \end{aligned}$$

28 $$\begin{aligned} \Phi _n({\varvec{r}}_{i}) ={}&{1\over 2} \sum ^{N/2^{nD}}_{k\ne i} 2^{nD}\epsilon f\Big (\frac{r_{i,k}-2^n r_o}{2^n\sigma }\Big ) +{1\over 2}\sum ^{nn}_{<k,i>}\frac{2^{nD}-1}{2^D-1}s\Big ({{\varvec{r}}_{i,k}\over 2^n}\Big )k_BT . \end{aligned}$$

It should be noted that the 2*l*-th spatial derivative of *s* in the interaction, $s^{(2l)}$, disappears when calculating $v_f$. The *D*-dimensional Hamiltonian after the *n* renormalization transforms $H_n$ is given as:

29 $$\begin{aligned} H_n ={}&\sum _{i=1}^{N/\lambda ^{D}}\Big \{ \frac{{p}_i^2}{2\lambda ^{D}m} +\Phi _n({\varvec{r}}_i) \Big \} \end{aligned}$$

30 $$\begin{aligned} \Phi _n({\varvec{r}}_i) ={}&{1\over 2}\sum _{k\ne i}\lambda ^{D}\epsilon f\Big (\frac{r_{i,k}-\lambda r_{o}}{\lambda \sigma }\Big ) +{1\over 2}\sum ^{nn}_{<k,i>}\frac{\lambda ^{D}-1}{2^D-1}s\Big ({{\varvec{r}}_{i,k}\over \lambda }\Big )k_BT \end{aligned}$$

where $\lambda =2^n$ is a scaling factor and $r_{i,k}=\left| {\varvec{r}}_i-{\varvec{r}}_k\right|$. $<k,i>$ denotes taking a summation of *k* on the nearest neighbor particles around the particle *i*. The second term of the right hand side of Eq. (30) has a contribution of $(k_B T / \epsilon ) s / (2^D-1) < (k_B T / \epsilon )/\{2(2^D-1)\}$ as $n\rightarrow \infty$. At $D = 3$ and $k_B T / \epsilon \simeq 1$, it is negligible comparing to the first term.

Finally, the equation of motion of the *i*-th particle is obtained by substituting the renormalized Hamiltonian Eqs. (29) and (30) into the canonical equation:

31 $$\begin{aligned} \frac{d {\varvec{p}}_i}{d t}={}&- {\lambda ^{D}\epsilon } \sum _{k\ne i}^{N/\lambda ^D}\frac{\partial }{\partial r_{i,k}} f\Big (\frac{r_{i,k}-\lambda r_o}{\lambda \sigma }\Big ) \frac{{\varvec{r}}_i-{\varvec{r}}_k}{r_{i,k}} -k_BT\sum ^{nn}_{<k,i>}\frac{\lambda ^{D}-1}{2^D-1} \frac{\partial }{\partial r_{i,k}}s\Big ({{\varvec{r}}_{i,k}\over \lambda }\Big )\frac{{\varvec{r}}_i-{\varvec{r}}_k}{r_{i,k}}, \end{aligned}$$

32 $$\begin{aligned} \dot{{\varvec{r}}}_i ={}&\frac{{\varvec{p}}_i}{\lambda ^D m}. \end{aligned}$$

### Scale transformation rule of physical properties

We derive scale transformation rules for three dimensional systems. Some physical properties are relevant and require scaling. For example, surface tension $\gamma$ can be expressed as $\gamma \sim \epsilon /r_o^{2}$^25^ [Sect. 4.3], thus it is scaled as $\gamma '= \lambda \gamma$. In a concept of local equilibrium, viscosity of fluid $\eta$ can be expressed as $\eta \sim \rho c_s r_o$^25^ [Sects. 1.16, 4.4], so it is scaled as $\eta '=\lambda \eta$. Hence, Reynolds number *Re* is transformed as $Re'=\lambda ^{-1} Re$, thus computational cost of high Reynolds number problems cannot be improved by RMD. However, RMD has an important advantage that it does not have the numerical viscosity and dispersion. The scale transformation rules of some physical properties and representative dimensionless parameters are tabulated on Table 1.

**Table 1:** Scale transformation rules (a) physical properties (b) the similarity law. Some dimensionless parameters are relevant.

(a)			(b)	
Physical property	Expression^25^	Transform	Number	Transforms
$\rho _N$ (number density)	$\sim 1/r_o^D$	$\rho _N'=\lambda ^{-D}\rho _N$	*Fr*(Froude)	$Fr'=Fr$
$\rho$ (mass density)	$\sim m /r_o^D$	$\rho '=\rho$	*Ca*(Capillary)	$Ca'=\lambda ^{3-D}Ca$
$c_s$ (sound velocity)	$\sim \sqrt{\epsilon r_o^2/(m\sigma ^2)}$	$c_s'=c_s$	*Nu*(Nusselt)	$Nu'=Nu$
*Y* (Young modulus)	$\sim {\rho c_s^2}$	$Y'=Y$	*Ma*(Mach)	$Ma'=Ma$
$\gamma$ (surface tension)	$\sim \epsilon /r_o^{2}$	$\gamma '=\lambda ^{D-2}\gamma$	*Pr*(Prandtl)	$Pr'=Pr$
$D_f$ (diffusion coefficient)	$\sim c_s r_o$	$D'_f=\lambda D_f$	*Bo*(Bond)	$Bo'=\lambda ^{2-D}Bo$
$\eta$ (viscosity)	$\sim \rho c_s r_o$	$\eta '=\lambda \eta$	*Re*(Reynolds)	$Re'=\lambda ^{-1}Re$
$\kappa$ (thermal conductivity)	$\sim \rho _N c_sr_o$	$\kappa '=\lambda ^{1-D}\kappa$	*De*(Deborah)	$De'=\lambda De$
$C_v$ (heat capacity)	$\sim \rho _N$	$C'_v=\lambda ^{-D}C_v$	*Kn*(Knudsen)	$Kn'=\lambda Kn$

### Renormalization of Langevin dynamics

We discuss an application of the RNG to dissipative dynamics. Renormalization group of the dissipative dynamics is derived. The damping coefficient $\xi$ can be expressed as $\xi \sim \eta r_o$, so it is scaled as $\xi \rightarrow \lambda ^{2} \xi$ by the renormalization. Hence, the RNG for the dissipative dynamics system ${\varvec{R}}_d({\varvec{K}})$ can be constructed by adding the transform $\xi \rightarrow \lambda ^{2} \xi$ into the renormalization transforms ${\varvec{R}}_b({\varvec{K}})$ as:

$$\begin{aligned} {\varvec{R}}_d({\varvec{K}})=(\lambda ^{-D}\beta , \lambda ^D \epsilon , \lambda r_o, \lambda \sigma , \lambda ^Dm, \lambda ^{2} \xi ) \end{aligned}$$

Renormalization of dissipative particle dynamics (DPD)^26^ is derived as follows:

33 $$\begin{aligned}\lambda ^D m\frac{d{\varvec{\dot{r}}}_i}{dt}=-\frac{\partial }{\partial {\varvec{r}}_i}\Phi _n({\varvec{r}}_{i}) -\sum _j \lambda ^{2} \xi {w}_D\Bigl ({r_{i,j}\over \lambda }\Bigr )({\varvec{e}}_{ij}\cdot {\varvec{\dot{r}}}_{i,j}){\varvec{e}}_{ij} +\sum _j\zeta _{ij}\sqrt{{w}_D\Bigl ({r_{i,j}\over \lambda }\Bigr )}~{\varvec{e}}_{ij} \end{aligned}$$

34 $$\begin{aligned}\left\{ \begin{array}{ll} \langle \zeta _{ij}(t)\rangle =0 &{}\\ \langle \zeta _{ij}(t)\zeta _{i'j'}(t')\rangle =2 \lambda ^{D+2} \xi k_B T (\delta _{ii'}\delta _{jj'}+\delta _{ij'}\delta _{ji'})\delta (t-t')&{}\\ \end{array}\right. \end{aligned}$$

Here, ${\varvec{e_{ij}}}={\varvec{r}}_{i,j}/r_{i,j}$,$w_D$ is a weighting function and $\zeta _{ij}(t)$ is $\delta$-correlated Gaussian noise. It can be easily verified that it follows the fluctuation dissipation theorem after the renormalization. In DPD, $\phi (r_{i,j})$ of Eq. (30) is defined as an interaction potential acting between particles in the dissipative dynamics system. To have the RNG constructed, it is required that the interaction potential as an identity element does not cause divergence on calculation of a free volume $v_f$ (Eq. (10)). For example, the Morse and LJ type potentials are renormalizable. A power-law repulsive potential $\phi =\epsilon (\sigma /(r_{i,j}-r_o))^{m}$ is also renormalizable because $\phi ^{(2l)}(r_{i,j})>0$ and $s(r_{i,j})\approx {1\over 2}$. However, the attractive potential $\phi =-\epsilon (\sigma /(r_{i,j}-r_o))^{m}$ can not be renormalized.

The relaxation time $\tau _{relax}\sim \xi /(r_oY)$ is scaled as $\tau '_{relax}=\lambda \tau _{relax}$. Since the Deborah number *De* of DPD is scaled as $De' =\lambda De$, *De* is at a fixed point only in the limitation of $De\rightarrow 0$ or $De\rightarrow \infty$. Therefore, *De* of renormalized DPD cannot be the same as that of the real system. On the other hand, renormalized DPD is equivalent to RMD in isothermal systems, provided that $De\ll 1$ and $\phi$ is an interatomic potential.

## Results

### Melting of an aluminum slab

**Figure 2:**
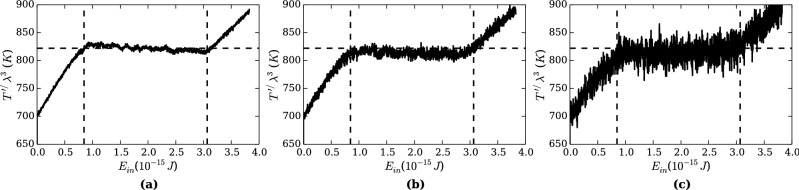
Temperature $T'/\lambda ^3$ versus total energy input to the slab $E'_{in}$. A horizontal dashed line is fitted to a plateau of the profile of the original system. Vertical dashed lines indicate endpoints of the plateau. (**a**) The original system ($\lambda = 2^0$). (**b**) The renormalized system with $\lambda = 2^1$. **c** $\lambda = 2^2$. The same dashed lines are shown on (**b**) and (**c**) to compare with the case (**a**). The renormalized systems show similar profiles, and the plateaus are approximately at the same temperature and the energy input as the original system. Temperature fluctuation increases as the system is renormalized due to the fewer number of particles.

In this section, we present verifications of RMD method by conducting two test problems. For the first problem, we considered melting of aluminum by simulating a slab of aluminum with heat added from its bottom. On Fig. 2a, temperature profile of the original system ($\lambda = 2^0$) is plotted along $E_{in}$, total energy input to the system. A presence of a plateau between two linear lines indicates the latent heat of melting. Three linear curves were fitted to the linear lines and the plateau of the profile by the least squares method to determine the melting temperature and the latent heat. These values were found to be 833.0 K and 467.3 kJ/kg, respectively. A horizontal dashed line on Fig. 2a indicates the melting temperature, and vertical dashed lines are endpoints of the plateau.

**Figure 3:**
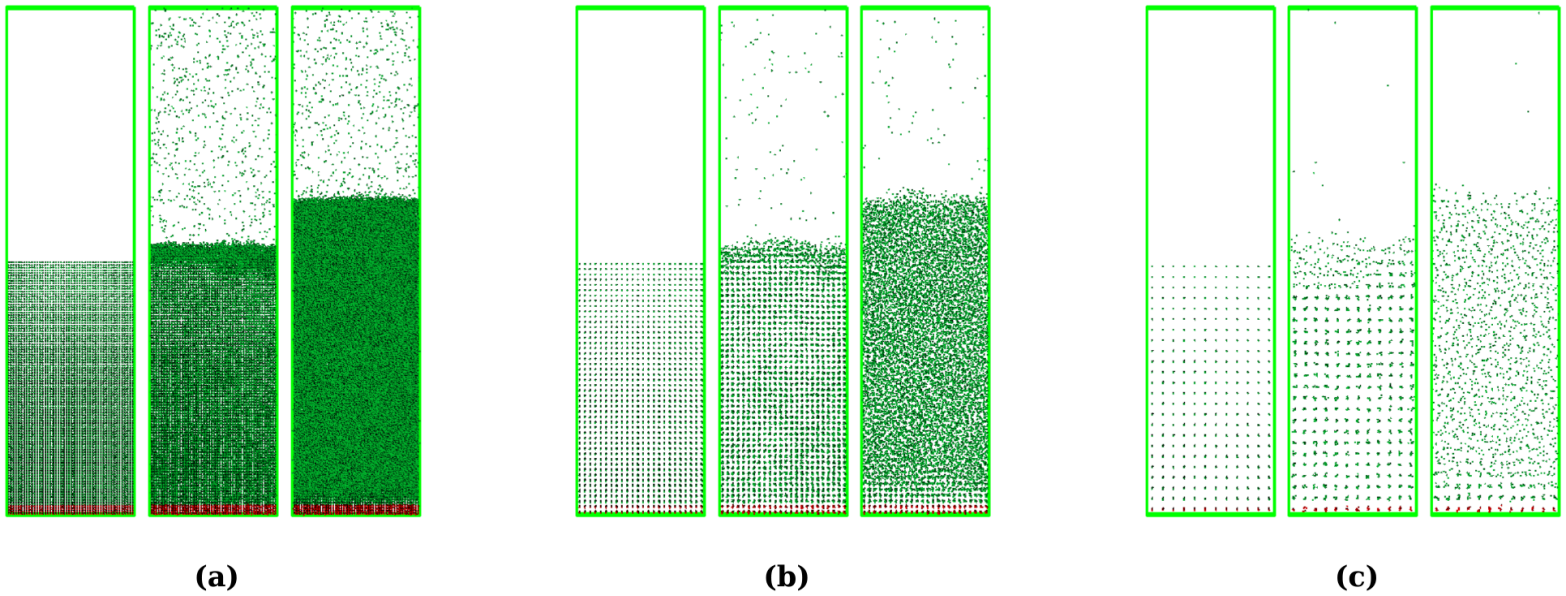
Snapshots of the melting slab. Green dots are aluminum atoms, and red dots are wall atoms on which the energy is added via the velocity rescaling. (**a**) The original system ($\lambda = 2^0$). (**b**) The renormalized system with $\lambda = 2^1$. (**c**) $\lambda = 2^2$. Left) Initial state. All the particles form a crystalline structure, which indicates that the slab is solid. (Middle) The slabs start to melt from surfaces. Liquid regions of three cases have approximately the same thickness. (Right) The solid regions almost completely melted. Thickness of the melted slabs remain the same for all three cases.

Simulations were repeated in renormalized systems at scaling factors of $\lambda = 2^1$ and $2^2$. The temperature profiles on Fig. 2b, c show the similar plateaus at the same temperature and energy input as the case of $\lambda = 2^0$, except that larger fluctuations are present due to the fewer particles in the renormalized systems. The similarity between the original and the renormalized systems, such as crystal structures and progress of melting surfaces, can be visually observed on snapshots shown on Fig. 3 while the particles are drastically coarsened.

### Collision of aluminum spheres

**Figure 4:**
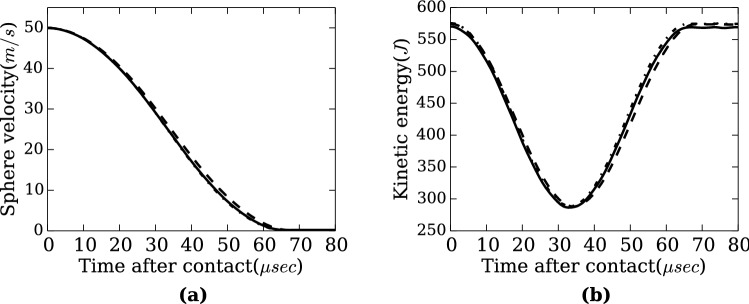
(**a**) Velocity profiles of the moving sphere versus time after contact. The sphere slows down and eventually reaches stationary through the contact, indicating that the collision is elastic. (**b**) Kinetic energy of the system versus time after contact. The kinetic energy is reduced through the contact and increased back to the initial value. Along with the velocity profile, it shows the kinetic energy of the moving sphere is transferred to the stationary sphere, which is another indication of the elastic collision. (Solid line) $\lambda = 2^{22}$, (dashed line) $\lambda = 2^{23}$, (half dashed line) $\lambda = 2^{24}$. The profiles of all the three cases are nearly indistinguishable. This would suggest the similarity of the phenomena by the renormalization.

For the next verification, we carried out a simulation of a collision of two aluminum spheres. The simulations were conducted with rather large scaling factors, $\lambda = 2^{22}$, $2^{23}$ and $2^{24}$ to investigate applicability of RMD to macroscopic systems. An aluminum sphere with radius of 34.3 mm and velocity of 50.0 m/s was collided to another stationary sphere with identical size and temperature, and behavior of the collision was monitored through sphere velocity and system kinetic energy. Figure 4a is a plot of the velocity of the moving sphere versus time after contact, and Fig. 4b shows profiles of the system kinetic energy. The test conditions and the simulated contact durations are tabulated on Table 2. For reference, a theoretical contact duration of elastic spheres^27^ [Sect. 1–9] is calculated to be $93.24 \times 10^{-6}$ s.

The kinetic energy and velocity profiles as well as the contact durations of three cases are agreed well whilst the number of particles are drastically reduced as the system is renormalized. This indicates that the similarity of the phenomena is retained after the renormalization in the same way as the melting of the slab, therefore suggests that the macroscopic MD calculation with only a few hundred thousands particles is achievable. It should be also noted that the total energy of each system is conserved throughout the calculations.

**Table 2:** Test conditions and the contact durations of the sphere collision. As the system is renormalized, the number of particles are reduced and the time step is increased accordingly to the scale transformation rule, whereas the contact durations remain relatively the same.

Scaling factor $\lambda$	$2^{22}$	$2^{23}$	$2^{24}$
Number of particles per sphere	137,729	17,357	2,171
Time step $\Delta t'$ ($10^{-8}$ s)	2.097	4.194	8.389
Contact duration ($10^{-6}$ s)	66.69	68.79	66.27

### Computational efficiency

**Figure 5:**
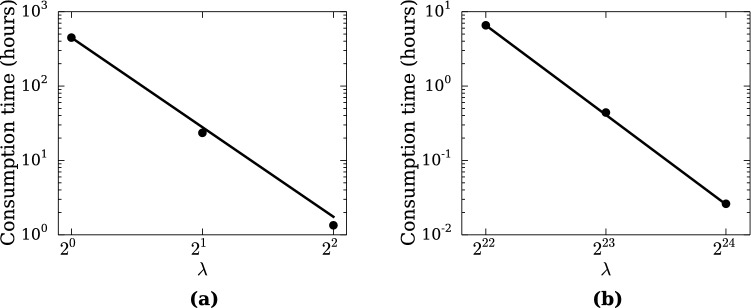
Consumption time of the RMD calculation versus the scaling factor. Black dots show the simulation results. Solid lines indicate the scale transformation rule $t'_c = t_c /2^{n(D+1)}$. (**a**) Melting of aluminum slab. (**b**) Collision of spheres. Both plots show that reduction of the consumption time follows the scale transformation rules.

Consumption time $t_c$ of MD simulation depends on the number of particles and the time step $\Delta t$. Since the time step of RMD is transformed as $\Delta t'\sim r'_o/c'_s\sim \lambda \Delta t$, combining with $N' \sim N/\lambda ^D$, $t_c$ is transformed as $t'_c = t_c/\lambda ^{D+1}$. Therefore, RMD achieves a higher efficiency by a factor of $\lambda ^{D+1}$ over conventional MD. Figure 5 shows the total consumption time versus the scaling factors. The plots show a clear indication that the computational efficiency improves accordingly as the system is renormalized.

It should be noted, however, that a term $s({\varvec{r}})$ resulted from the renormalization is neglected in the current test problems. If the term is not negligible, such as at a high temperature condition $k_BT\gg \epsilon$ or in the lower dimensions $D<3$, $s({\varvec{r}})$ needs to be included to calculations so that it would result in additional computational cost. In MD calculations, the largest portion of the computational cost is calculations of forces. A simple estimation suggests that, in the worst case scenario, the practical computational efficiency would be approximated as a factor of $2^{n(D+1)-1}$, because only the calculations of forces are doubled.

DPD contains a time constant $\tau =m/\xi$. The time step of DPD is also restricted by $\Delta t' \ll \tau '$ and $\tau ' = \lambda \tau$. This implies that the time step $\Delta t$ is scaled as $\Delta t'=\lambda \Delta t$, therefore it yields the same computational efficiency as RMD.

### Critical behavior in the liquid-vapor region on mesoscale

We investigate the applicability of the renormalized Hamiltonian at a fixed point to phase transition near the critical point. To do so, we observe the behavior of specific heat at constant volume $C_V$ in the near-critical region by the finite size scaling^28,29^ [Sect. V.2.2]. When scaling of the distribution function by the renormalization is invariant near the critical point, following the finite size scaling theory, $C_V$ and *T* can be scaled as:

35 $$\begin{aligned} \Psi (x)&= { \frac{L^{D-2/\nu }\lambda ^D}{(xL^{-1/\nu }+1)^2} C'_V ((xL^{-1/\nu }+1)T'_c)} \end{aligned}$$

36 $$\begin{aligned} x&=L^{1/\nu }\frac{T'-T'_c}{T'_c} \end{aligned}$$

where $T_c$ is critical temperature, $\nu$ is critical exponent and *L* is domain size (side length of a cubic domain). Although $\Psi (x)$ is a function of a variable *x*, a form of the function $\Psi (x)$ of x is not explicitly known. However, $\Psi (x)$ obtained from different system sizes can be collapsed onto a single curve if values of $T_c$ and $\nu$ are valid.

**Figure 6:**
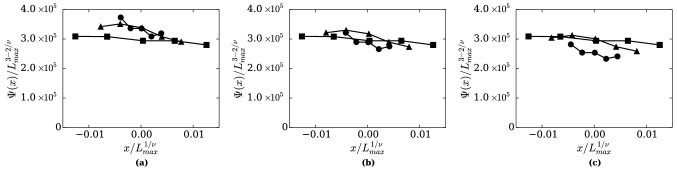
$\Psi (x) / L^{3-2/\nu }_{max}$ versus $x / L^{1/\nu }_{max}$. (**a**) $\nu = 0.59$, (**b**) $\nu = 0.63$, (**c**) $\nu = 0.67$. $\bullet$: $L = 11\sigma '$, $\blacktriangle$: $L = 16.5\sigma '$, $\blacksquare$: $L = 22\sigma '$. $\Psi (x) / L^{3-2/\nu }_{max}$ from the different system sizes roughly collapse onto the single curve at $\nu = 0.63$ whereas curves do not overlap in other two cases.

We calculated $C'_V$ of argon near the critical temperature in three different mesoscale sizes, $L = 11\sigma ', 16.5\sigma '$ and $22\sigma '$ by RMD. The scaling factor is set to $\lambda =2^8$. $\Psi (x)$ and *x* are calculated by Eqs. (35) and (36) from the resulting $C'_V$ and its temperature dependence. We explored several values of the critical exponent $\nu$ for the critical temperature, $T_c' / \lambda ^3 = 157.2 K$^30–32^ and located that $\Psi (x)$ of the different mesoscale sizes approximately collapse onto a single curve at $\nu = 0.63\pm 0.04$ as shown Fig. 6. Figure 6 is a plot of $\Psi (x)/L^{3-2/\nu }_{max}$ versus $x/L^{1/\nu }_{max}$ at $\nu$ = 0.59, 0.63 and 0.67, where $L_{max}$ refers to the largest one of three sizes, i.e. $L_{max}=22\sigma '$. $\nu$ = 0.62 and 0.64 were also explored, and the curves of three sizes were not clearly distinguishable (the plots are omitted). Therefore, we found that the curves of three sizes overlap at $\nu = 0.63\pm 0.01$. This is close to the value of the existing work $\nu = 0.630$ obtained by the conventional MD calculation of the LJ fluid^33^ and by the study of the same universality class (i.e. three dimensional Ising model)^34,35^. More precise extraction of the critical exponent is not a scope of the current work, however, our results demonstrated that RMD is capable of reproducing the critical behavior. It should be mentioned that the critical slowing down is not improved due to the reduction in the number of particles by the renormalization.

## Discussion

In this study, we have constructed the RNG of MD by using the Migdal–Kadanoff approximation. The total mass and energy are conserved in the renormalized systems, and the obtained scale transformation rules show that especially Young modulus and speed of sound remain invariant. We conducted two simulations to validate the RMD method. Both problems have demonstrated that the expected values of physical quantities are in good agreement after the renormalization, and the computational efficiency is improved as expected by $2^{n(D+1)}$ over conventional MD. Furthermore, to observe behavior of RMD near the critical region, temperature dependence of $C_V$ of the LJ fluids was calculated near the critical temperature. The results showed that the critical exponent $\nu$ can be extracted by the finite size scaling theory in RMD, and it was confirmed that the obtained $\nu$ was agreed with previous work regarding the conventional MD calculation of the LJ fluid and the study of the same universality class (i.e. three dimensional Ising model). However, RMD does not improve the critical slowing down due to the reduction in the number of particles by the renormalization.

In addition, we explored the application of the RNG to DPD, and derived the renormalized DPD equations. To have the RNG constructed for DPD, it is required that an interaction potential as an identity element does not cause divergence on calculation of a free volume $v_f$ (Eq. (10)). Renormalized DPD is equivalent to RMD in isothermal systems, provided that an interaction $\phi$ is an interatomic potential and the Deborah number $De\ll 1$. We also showed that the computational efficiency of renormalized DPD is similar to that of RMD.

Our work proposes a new approach for MD, which provides a drastic improvement on the computational efficiency for large scale systems while retaining the advantages of conventional MD. For future work, we will also investigate and discuss how the term of *s*(*r*) would contribute to the RMD calculations in the conditions such that the term is not negligible. Starting from the current study, which coarse-grains the whole domain uniformly, we would extend RMD to more practical method, such as the derivation of the local group which should be applicable in a similar manner to irregular meshes.

## Methods

All the simulations of the current work are carried out by our in-house code written in C. Practically, computational algorithms of RMD is identical to conventional MD, which solve the equations of motion Eqs. (31) and (32), except that the temperature T, the potential parameters $\sigma$, $r_o$, $\epsilon$, and the mass *m* need to be scaled accordingly to the renormalization transforms ${\varvec{K}}_n = (2^{-nD}\beta ,2^{nD}\epsilon ,2^n r_o,2^n\sigma , 2^{nD} m)$ (Eq. (27)). Second term of the right hand side of Eq. (31) is neglected in the current work. The velocity Verlet scheme is used for the time integration.

### Test conditions of the melting phenomenon

The Morse potential is adopted for the potential of aluminum atoms:

37 $$\begin{aligned} \phi '({\varvec{r}})=\epsilon ' \Big \{e^{-2\big ({r-r'_o \over \sigma '}\big )} -2e^{-\big ({r-r'_o \over \sigma '}\big )}\Big \}, \end{aligned}$$

where $\epsilon ' / \lambda ^3 = 1.92 \times 10^{-20}$ J, $\sigma ' / \lambda = 4.255\times 10^{-11}$ m, $r'_o / \lambda = 0.286 \times 10^{-9}$ m, and $m' / \lambda ^3 = 4.48 \times 10^{-26}$ kg. The potential is cut-off at $r_c = 3.8 r'_o$ to reduce the computational cost. Time step size is $\Delta t' / \lambda = 5.0 \times 10^{-15}$ s.

Particles are initially placed in fcc configuration with $48/\lambda \times 24/\lambda \times 24/\lambda$ cells, which gives the total number of particles $N_p'\lambda ^3 = 110{,}592$ and initial thickness of the slab $1.94 \times 10^{-8}$ m. Computational domain size is $(L_x, L_y, L_z) = (3.883 \times 10^{-6}\, \text {m}, 0.971 \times 10^{-6}\, \text {m}, 0.971 \times 10^{-6}\, \text {m})$. Note that $L_x$ is set to be twice the initial thickness of the slab so that it provides some space for the slab to expand and evaporate on the surface. Mirror boundary conditions are applied in the x-direction to prevent atoms from leaving the domain, and periodic boundary conditions are applied to all other directions.

Four layers of the atoms from the bottom of the domain are treated as a wall for heat transfer (shown as red particles on Fig. 3). The number of wall atoms is $N'_{p,wall}\lambda ^3=4608$. The heat is added to the slab by increasing the kinetic energy of the atoms of the wall via rescaling the momentum. Only momentum of each particle in the wall is rescaled as ${\varvec{p}}_{i, rescaled} = {\varvec{p}}_i \sqrt{1 + 2m'\Delta e'_i / p^2_i}$, where ${\varvec{p}}_i$ is the current momentum of particle *i*, ${\varvec{p}}_{i, rescaled}$ is the momentum after rescaling and $\Delta e'_i$ is energy added to the particle at every $4.0 \times 10^{-13}$ s. This interval is commonly used for all $\lambda = 2^0, 2^1$ and $2^2$. The energy $\Delta E' = N'_{p,wall} \Delta e'_i = 7.68 \times 10^-{20}$ J is the total energy added to the wall. The wall atoms interact with other atoms via the Morse potential, and spring-like forces also act on these atoms. This allow the wall atoms to vibrate but not freely move away from their original positions. The spring-like force is given as ${\varvec{F}}'_{i,spring} = -k' \delta {\varvec{r}}_{i}$ where $k' /\lambda = 20.0 \, \hbox {kg/s}^2$ is a spring constant and $\delta {\varvec{r}}_{i}$ is a displacement of an atom *i* relative to its original position.

Temperature is initially set as $T' / \lambda ^3 = 700$ K, and is relaxed at the same temperature for the first $1.0 \times 10^{-6}$ s by the Berendsen thermostat. The thermostat is applied at every $4.0 \times 10^{-13}$ s, and the time constant of the thermostat is set to $1.0 \times 10^{-5}$ s. This interval and duration are commonly used for all $\lambda =2^0$, $2^1$ and $2^2$. Heat addition is then started and the temperature of the slab is sampled at every 2,000 time steps. The wall particles are not included to the temperature calculation. Note that the total energy added to the system is invariant, $\Delta E' = \Delta E$, but since the number of the wall atoms are reduced as $N'_{p,wall} \lambda ^3 = N_{p,wall}$, the energy added to each atom is scaled as $\Delta e'_i / \lambda ^3 = \Delta e_i$.

### Test conditions of the collision of two sphere

The potential and parameters are identical to the first problem, except that the potential is constant $\phi (r_o)$ if $r > r_o$ for interactions between particles which belong to different spheres. This provides only repulsive forces between spheres to prevent spheres from sticking together upon contact.

Particles are initially placed in fcc configuration inside a spherical region of a given radius $R = 3.863 \times 10^{-2}$ m. The radius is selected so that particles forming sphere surface are located on the exactly same distance from the center of the sphere at all three cases. Temperature is initially set to be $T' / \lambda ^3 = 100$ K, and gradually cooled down to 0 K by the weak Berendsen thermostat. The time constant of the thermostat is set to $20.0 \Delta t'$. The thermostat is applied every $2.19 \times 10^{-6}$ s, and its duration is $1.47 \times 10^{-3}$ s. This interval and duration are commonly used for all $\lambda =2^{22}$, $2^{23}$ and $2^{24}$.

The obtained sphere is duplicated and placed so that separation distance of sphere centers is $7.49 \times 10^{-2}$ m. The spheres are left stationary (without thermostat) for another $3.66 \times 10^{-4}$ s for relaxation, then velocities of the particles of one sphere are set to be 50.0 m/s toward the other sphere. The velocity of the sphere is calculated by averaging the velocities of the particles which belong to the sphere.

### Test conditions of the specific heat calculation

The Lennard-Jones potential is adopted for the potential of argon atoms:

38 $$\begin{aligned} \phi '({\varvec{r}})=4\epsilon ' \Big \{\Big ({\sigma '\over r}\Big )^{12} -\Big ({\sigma ' \over r}\Big )^6\Big \}, \end{aligned}$$

where $\epsilon ' / \lambda ^3 = 1.654 \times 10^{-21}$ J, $\sigma ' / \lambda = 3.405\times 10^{-10}$ m and $m' / \lambda ^3 = 6.634 \times 10^{-26}$ kg. Time step size $\Delta t' / \lambda = 1.0 \times 10^{-15}$ s, cut-off distance of the potential range $r'_c = 5.0 \sigma '$ and the scaling factor $\lambda = 2^8$ are employed. The computational domain is cubic with a side length of *L*, and periodic boundary conditions are imposed on all surfaces.

To observe the behavior in near-critical region by the finite size scaling, three different domain sizes are employed; $L = 11\sigma '$, $16.5\sigma '$, $22\sigma '$. For all domain sizes, mass density is fixed at the critical density of argon $\rho _c = 534.1\,\hbox {kg/m}^3$^36^, thus the corresponding number of particles are $N' = 423$, 1428, 3386, respectively. $C'_V$ are calculated at $T'/\lambda ^3 = 155, 156, 157, 158 \, and \, 159$ K, which is around the critical temperature of argon, $T_c'/\lambda ^3 = 157.2$ K^30–32^.

The temperature is initially set at $T'/\lambda ^3 = 360$ K, then the Nose–Hoover thermostat is turned on at $t = 5.12\times 10^{-9}$ s to bring the system to the target temperature. The total energy is sampled from $t = 5.12\times 10^{-7}$ to $1.02\times 10^{-6}$ s at every 100 time steps. $C'_V$ is calculated from the fluctuation of the total energy $E'$ under the NVT condition as follows:

39 $$\begin{aligned} C'_V = \frac{\langle E'^2 \rangle - \langle E' \rangle ^2}{L^3 k_B T'^2} \end{aligned}$$

where $\langle \rangle$ indicates the ensemble average. Note that $C_V$ is scaled as $C_V' = C_V / \lambda ^3$ as shown on Table 1a. Fluctuations of the energy near the critical point is highly sensitive to initial configurations of the system. To obtain statistically more meaningful results, we averaged over the results of five runs with different initial configurations for each test condition.

## Data Availability

The data that support the findings of this study are available from the corresponding author upon request.

## Code availability

An in-house MD computer code was used for all the calculations of this study. The code is available from the corresponding author upon request.
